# The limits of bio-molecular modeling with large language models: a cross-scale evaluation

**DOI:** 10.1093/bioinformatics/btag550

**Published:** 2026-07-23

**Authors:** Yaxin Xu, Yue Zhou, Tianyu Zhao, Zhengyu Ma, Fengwei An, Zhixiang Ren

**Affiliations:** College of Engineering, Southern University of Science and Technology, Shenzhen 518055, China; Department of Advanced Computing, Pengcheng Laboratory, Shenzhen 518055, China; Department of Advanced Computing, Pengcheng Laboratory, Shenzhen 518055, China; LingQiong AI Lab, Shanghai Smart Logic Technology Co., Ltd, Shanghai 200443, China; Department of Advanced Computing, Pengcheng Laboratory, Shenzhen 518055, China; School of AI for Science, Peking University Shenzhen Graduate School, Shenzhen 518055, China; College of Engineering, Southern University of Science and Technology, Shenzhen 518055, China; LingQiong AI Lab, Shanghai Smart Logic Technology Co., Ltd, Shanghai 200443, China

## Abstract

**Motivation:**

The modeling of bio-molecular system across molecular scales remains a central challenge in scientific research. Large language models (LLMs) are increasingly applied to bio-molecular discovery, yet systematic evaluation across multi-scale biological problems and rigorous assessment of their tool-augmented capabilities remain limited.

**Results:**

We reveal a systematic gap between LLM performance and mechanistic understanding through the proposed cross-scale bio-molecular benchmark: BioMol-LLM-Bench, a unified framework comprising 26 downstream tasks that covers 4 distinct difficulty levels, and computational tools are integrated for a more comprehensive evaluation. Evaluation on 13 representative models reveals 4 benchmark-specific observations: chain-of-thought-style training does not consistently improve performance on the evaluated biological tasks; the evaluated hybrid mamba–attention model shows strong performance on long bio-molecular sequence tasks; supervised fine-tuned models show task-specific specialization with reduced performance in some general settings; and current LLMs perform better on classification tasks than on challenging regression tasks under this benchmark setting.

**Availability:**

Source code is available at https://github.com/AI-HPC-Research-Team/BioMol-LLM-Bench

## 1 Introduction

Bio-molecule plays a foundational role across a wide range of biological and chemical domains (Kortemme and Baker 2004, Gibson *et al*. 2008, Powner *et al*. 2009). Multi-scale bio-molecular modeling has emerged as a critical paradigm, aiming to bridge different levels of representation from monomer-level molecule to polymer complex. They underpin applications from bio-materials design to drug delivery systems (Langer and Tirrell 2004, Douglas *et al.* 2012). However, effectively integrating and modeling information across these scales remains a significant challenge. With the rapid growth of data availability and computational power, there is an increasing reliance on large language models (LLMs) (Achiam *et al*. 2023, Bai *et al*. 2023, Gemini Team *et al*. 2023, Touvron *et al*. 2023, Abdin *et al*. 2024, Gemma Team *et al*. 2024, Basant *et al*. 2025, Guo *et al*. 2025) to accelerate property prediction and model complex interactions simultaneously. In bio-molecular sciences, these models (Beltagy *et al*. 2019, Bran *et al.* 2023) hold particular promise because they can reason about molecules, proteins, and their interactions in natural language, thereby enabling more intuitive exploration of chemical space. Recent years have witnessed a proliferation of domain-adapted models (Pei *et al*. 2024, Xia *et al*. 2025, Wang *et al.* 2025, Zhuang *et al*. 2025), ranging from general-purpose foundation models fine-tuned on scientific corpora to architectures specifically designed for bio-molecular understanding, each claiming varying degrees of proficiency on bio-molecular tasks. Notable examples include TxGemma Wang *et al.* (2025), which specializes in therapeutic tasks by processing diverse modalities such as small molecules and natural text to predict therapeutic properties.

Yet the rapid development of these models has outpaced our ability to systematically evaluate their capabilities. General-domain benchmarks (Saikh *et al*. 2022, Wang *et al*. 2023, 2024, Laurent *et al*. 2024, Olea *et al*. 2024, Rein *et al.* 2024, Sun *et al*. 2024, Phan *et al*. 2025) such as MMLU-Pro (Wang *et al.* 2024) and AI2ARC (Olea *et al.* 2024), while useful for assessing broad reasoning capabilities, lack the specialized knowledge required for meaningful evaluation on bio-molecular tasks. Conversely, domain-specific benchmarks (Walker *et al*. 2010, Wu *et al*. 2018, Rao *et al*. 2019, Dallago *et al*. 2021, Zhu *et al*. 2023, Shen *et al.* 2024, Yu *et al*. 2024) often focus on single task types such as molecular reaction outcome prediction, or protein function annotation, without considering how models perform across the interconnected scales of bio-molecular problems. These limitations obscure important patterns in model behavior: a model that excels at predicting small molecule solubility may fail catastrophically on protein-protein interaction tasks, such trade-offs remain poorly characterized. More significantly, current evaluation frameworks (Ding *et al*. 2025, Gao *et al.* 2025b) provide limited insight into how different model architectures and training approaches affect model behavior across cross-scale bio-molecular tasks.

Furthermore, LLMs are increasingly evolving into agentic systems with tool-use capabilities. Existing benchmarks (Sorkun *et al*. 2019, Huang *et al*. 2021, Thumuluri *et al*. 2022, Notin *et al.* 2023, Li *et al*. 2024, Zhao *et al*. 2025) lack integration with computational tools and are limited to task-oriented question answering. But in real-world scientific practice, researchers combine conceptual understanding with specialized software (Bran *et al*. 2023, Abramson *et al*. 2024, Swanson *et al*. 2024, van Kempen *et al*. 2024, Ding *et al*. 2025, Gao *et al.* 2025a) for bio-molecular modeling. The benchmarks mentioned above typically evaluate models under artificial constraints, requiring direct question answering or chain-of-thought (CoT) thinking, thereby failing to provide a comprehensive assessment of model capabilities.

**Figure 1:**
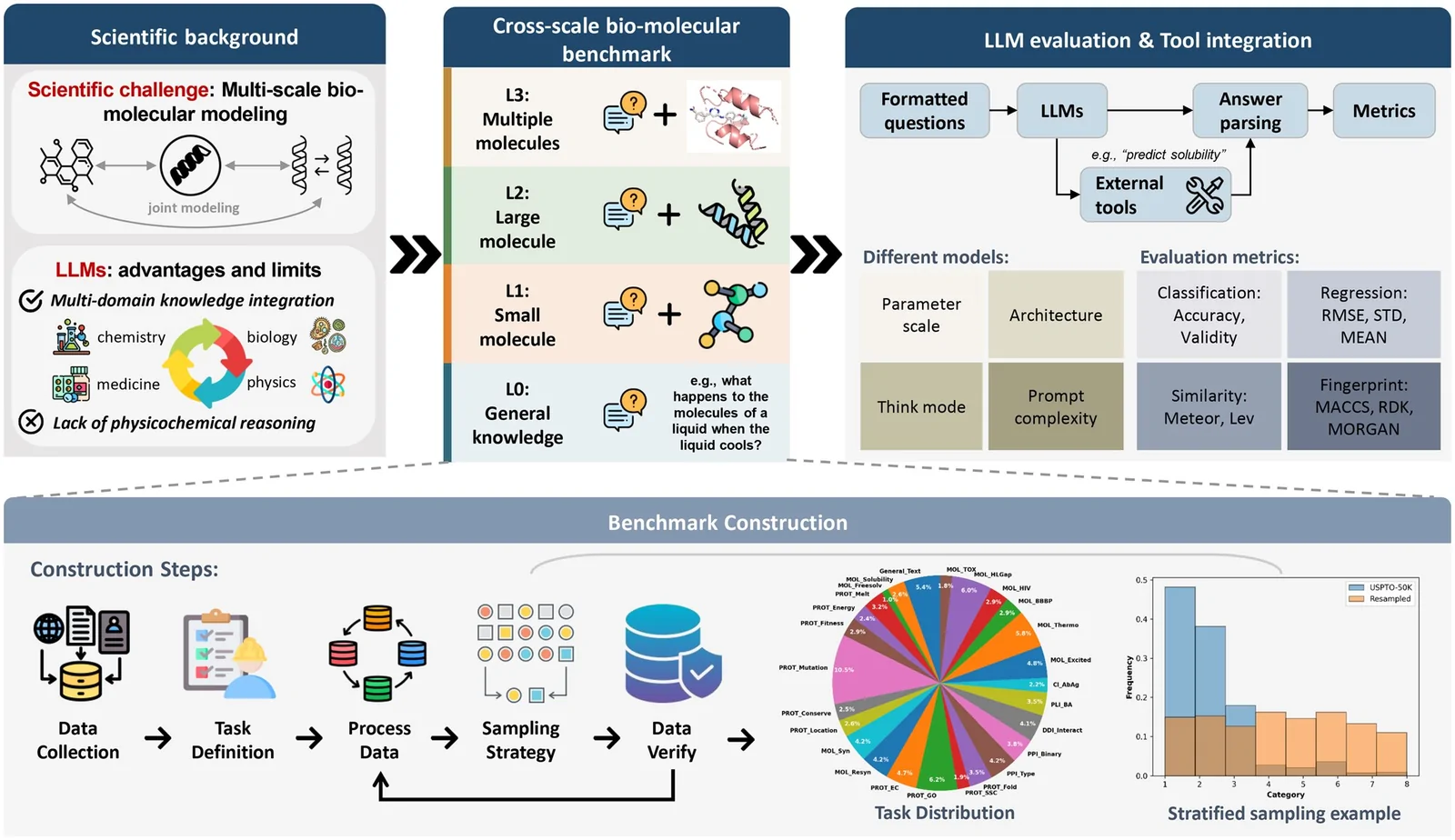
Overview of BioMol-LLM-Bench. The framework addresses limitations in existing benchmarks by incorporating specialized bio-chemistry tools and enabling cross-scale evaluation with 26 tasks across 4 levels. The evaluation pipeline integrates automatic answer extraction and metrics computation. 13 models are compared under different experiment settings.

To address these gaps, we introduce the cross-scale bio-molecular benchmark (BioMol-LLM-Bench), a comprehensive evaluation framework (Fig. 1) that aims to help us evaluate the capabilities of LLMs from the perspective of practical scientific applications. Our benchmark encompasses 4 hierarchical levels ($L_{0}$ to $L_{3}$) corresponding to increasing structural and functional complexity. This hierarchical organization enables fine-grained analysis of how model capabilities scale with problem complexity. We provide an automated evaluation pipeline that parses model outputs into required formats. Beyond traditional accuracy measures, the pipeline evaluates output validity, which determines whether model responses conform to required formats. Specialized computational tools are integrated into the evaluation framework. This allows evaluation of model ability in computational tool orchestrating and argument parsing.

Furthermore, we conducted experiments on 13 general-purpose and domain-specific models, revealing the benchmark’s discriminative power and utility in guiding future LLM design.

- *Training data:* On biological tasks, we found that CoT provides a slight improvement and may even weaken performance. The reasoning process appears sound in linguistics, but contains a self-contradictory description and chemical inconsistency.
- *Model Architecture:* Among the evaluated models, the hybrid mamba–attention model shows strong performance on bio-molecular sequence tasks involving long-range dependencies, and in several tasks outperforms larger transformer-based models.
- *Training strategy:* The evaluated supervised fine-tuned (SFT) models show task-specific strengths, but also reduced robustness on some general or out-of-distribution tasks.
- *Task performance:* Although LLMs perform well on several classification tasks, none of the evaluated models achieves meaningful performance on challenging regression tasks such as amino acid-level property prediction.

## 2 Results

### 2.1 Cross-scale bio-molecular benchmark

#### 2.1.1 Benchmark construction and data curation

The construction of BioMol-LLM-Bench followed a multi-stage pipeline designed to aggregate and refine high-quality bio-molecular data from heterogeneous sources including MMLU-Pro (Wang *et al.* 2024), USPTO-50K (Maziarz 2024), DrugBank (Knox *et al.* 2024), PDB (Berman *et al.* 2000). During the initial data processing phase, raw data were subjected to rigorous deduplication and structural validation to ensure the integrity of SMILES strings and FASTA sequences. This was further refined through a LLM verification process using a specialized prompt to assign confidence scores (0≤c≤1) to eliminate entries that unrelated to the bio-molecular field. Then, a hierarchical downstream task stratification was implemented, categorizing tasks into 4 levels ($L_{0}$ to $L_{3}$) according to input data type, ranging from general molecular text to specialized large-molecule complexes (Table 1). Finally, a stratified sampling strategy was applied to the curated dataset of each task to mitigate class imbalance and ensure a representative distribution across the bio-chemical space.

**Table 1:** Summary of benchmark tasks.

Level	Task (abbr.)	Inputs	#Num	Source
L0	General_Text	Text	226	GPQA_Diamond Rein *et al.* (2024), MMLU_Pro Wang *et al.* (2024), AI2ARC Olea *et al.* (2024)
L1	MOL_Solubility	Text, SMILES	109	AqSolDB Sorkun *et al.* (2019), esol Delaney (2004)
	MOL_Freesolv	Text, SMILES	43	Freesolv Mobley and Guthrie (2014)
	MOL_Syn	Text, SMILES	176	USPTO-50K Maziarz (2024)
	MOL_Resyn	Text, SMILES	176	USPTO-50K Maziarz (2024)
	MOL_Excited	Text, SMILES	200	QM8 Ramakrishnan *et al.* (2015)
	MOL_Thermo	Text, SMILES	240	QM9 Ruddigkeit *et al.* (2012)
	MOL_BBBP	Text, SMILES	119	BBBP Martins *et al.* (2012)
	MOL_HIV	Text, SMILES	120	MoleculeNet Wu *et al.* (2018)
	MOL_HLGap	Text, SMILES	251	PCQM4Mv2 Hu *et al.* (2021), QM9 Ruddigkeit *et al.* (2012)
	MOL_TOX	Text, SMILES	76	tox21 Wu *et al.* (2018)
L2	PROT_Melt	Text, AA-Seq.	133	Meltome_atlas Jarzab *et al.* (2020)
	PROT_Energy	Text, AA-Seq.	101	Tm262 Li *et al.* (2024), S669 Pancotti *et al.* (2022)
	PROT_Fitness	Text, AA-Seq.	122	GB1 Wu *et al.* (2016), avGFP Sarkisyan *et al.* (2016)
	PROT_Mutation	Text, AA-Seq.	436	ProteinGym Notin *et al.* (2023)
	PROT_Conserve	Text, AA-Seq.	104	VESPA Dallago *et al.* (2021)
	PROT_Location	Text, AA-Seq.	107	DeepLoc Thumuluri *et al.* (2022)
	PROT_EC	Text, AA-Seq.	196	DeepFRI Gligorijević *et al.* (2021)
	PROT_GO	Text, AA-Seq.	258	DeepFRI Gligorijević *et al.* (2021)
	PROT_SSC	Text, AA-Seq.	77	CB513 Zhou and Troyanskaya (2014), CASP12 Moult *et al.* (2018)
	PROT_Fold	Text, AA-Seq.	145	PDB Berman *et al.* (2000)
L3	PPI_Type	Text, Two AA-Seqs.	175	SHS27k Chen *et al.* (2019)
	PPI_Binary	Text, Two AA-Seqs.	159	TAGPPI Song *et al.* (2022)
	DDI_Interact	Text, Two SMILES	170	Drugbank Knox *et al.* (2024)
	PLI_BA	Text, AA-Seq., SMILES	146	SKEMPI Jankauskaitė *et al.* (2019), bindingdb Liu *et al.* (2025), davis Davis *et al.* (2011)
	CI_AbAg	Text, Three AA-Seqs.	90	AbBiBench Zhao *et al.* (2025)

The table presents 26 tasks categorized into 4 hierarchical levels (L0–L3) based on input complexity. For each task, the table specifies the task abbreviation, input modalities, number of test samples (#Num), and the original data sources. SMILES and AA-Seq. represent sequence strings of molecule and protein, respectively.

#### 2.1.2 Integration of computational tools

Current artificial intelligence models, particularly LLMs, possess varying degrees of capability in automatically invoking tools and parsing function parameters. To further enhance the evaluation capability of the benchmark for models, we integrated a suite of domain-specific tool interfaces (Gao *et al.* 2025a). This integration allows for an agentic workflow where models can invoke specific predictive functions, such as solubility_lipophilicity_hydration() and BBB_penetrance(), to derive ADMETAI (Absorption, Distribution, Metabolism, Excretion, Toxicity, and AI-predicted) properties.

#### 2.1.3 Evaluation framework and metrics

The final stage of the benchmark involves a standardized evaluation pipeline where formatted questions are processed by models under various experimental configurations. An automatic answer extraction method was proposed to parse model output, so that the extracted content could be used to compare with corresponding labels. For different downstream tasks, model performance is quantified using distinct metric clusters.

### 2.2 Comparative performance of LLMs

To demonstrate the utility and discriminability of our benchmark, we evaluated 13 LLMs (Table 2) using detailed prompt instructions (illustrated in Section 4), encompassing a range of architectural designs (dense and sparse, Transformer and Mamba), as well as both domain-specific and general-purpose models. Table 2 presents a comprehensive ranking of the models. The models are ranked from 1 (best) to 13 (worst) on each individual task, with the overall rank across all tasks summarized in the bottom row.

**Table 2:** Model rankings on each benchmark tasks.

Tasks	A	B	C	D	E	F	G	H	I	J	K	L	M
General_Text	3	11	8	5	7	10	9	1	12	13	4	6	2
PROT_EC	12	13	8	1	3	4	5	7	10	11	6	9	2
PROT_Location	11	13	7	1	5	2	4	10	6	12	3	9	8
PPI_Type	1	12	9	2	3	4	8	10	5	13	7	11	6
PPI_Binary	1	13	6	8	4	2	11	10	7	12	5	9	3
MOL_TOX	9	12	8	3	1	10	7	11	2	13	5	4	6
MOL_HIV	8	13	2	9	3	4	6	11	10	12	1	7	5
MOL_BBBP	1	13	7	3	5	8	10	11	6	12	9	4	2
PLI_BA	8	7	6	8	2	1	13	4	12	8	8	5	3
MOL_Thermo	7	11	8	9	3	2	13	1	12	10	4	5	6
MOL_Excited	7	9	1	4	5	11	13	3	12	6	8	10	2
PROT_Energy	3	8	6	2	1	11	13	4	12	10	9	5	7
MOL_Freesolv	6	8	2	3	4	10	13	1	12	7	11	9	5
MOL_HLGap	2	10	1	3	4	11	13	5	12	9	6	7	8
PROT_Mutation	7	4	8	6	3	10	13	1	12	11	9	2	5
MOL_Solubility	1	8	6	9	2	11	13	3	12	10	5	7	4
PROT_Fitness	4	5	6	7	2	11	13	1	12	10	9	3	8
PROT_Melt	5	4	7	9	1	11	13	2	12	10	6	3	8
CI_AbAg	1	10	9	6	2	11	13	3	12	8	5	7	4
PROT_GO	10	11	6	3	13	7	9	2	1	12	5	8	4
DDI_Interact	7	8	9	5	1	13	10	11	2	12	3	6	4
MOL_Syn	7	13	2	5	10	6	8	11	1	12	3	9	4
MOL_Resyn	5	10	2	4	8	1	7	12	9	11	3	13	6
**Overall Rank**	**4**	**11**	**5**	**3**	**1**	**9**	**12**	**7**	**10**	**13**	**6**	**8**	**2**

The overall rank is obtained by calculating the ranking of all models based on the average of each model’s rankings across all tasks. A: Phi-4-14B, B: GPT-oss-20B, C: Gemma-3-12B, D: Qwen-3-14B, E: NVIDIA-Nemotron-Nano-9B-v2, F: Mistral-Nemo-Instruct-2407, G: Llama-3.1-8B-Instruct, H: DeepSeek-R1-Distill-Qwen-14B, I: TxGemma-Chat-9B, J: NatureLM-8x7B, K: Qwen3-235b-a22b-2507, L: GPT-5-mini, M: DeepSeek-v3.1.

NVIDIA-Nemotron-Nano-9B-v2 emerges as the overall top performer with a composite rank of 1, demonstrating exceptional versatility across task categories. It achieves 4 first-place rankings among others. DeepSeek-V3.1 secures the second overall position, showing particularly strong performance on General_Text task. Moreover, several models exhibit domain-specific expertise. Phi-4-14B achieves first-place rankings on two L3-level tasks, suggesting particular strength in interaction prediction tasks. However, NatureLM-8x7B generally falls below average across tasks, contributing to its overall lower standing.

The ranking demonstrates that no single model dominates across all tasks. The strong performance of NVIDIA-Nemotron-Nano-9B-v2 within BioMol-LLM-Bench suggests that the combination of mamba and attention mechanisms may be beneficial for capturing global and local information in tasks involving long molecular SMILES strings or protein sequences.

The results of DeepSeek-V3.1 indicate that large-scale models with massive parameters, extensive data, advanced training strategies possess stronger knowledge retention capabilities compared to smaller models, thereby yielding broadly applicable representations. The consistent under-performance of Llama-3.1-8B-Instruct and NatureLM-8x7B indicates their potential limitations for bio-molecular tasks.

To further quantify the specialization–generalization trade-off associated with domain-specific fine-tuning, we summarized task-group average rankings for domain-specific fine-tuned models in Table S2, available as supplementary data at *Bioinformatics* online. The results show that TxGemma-Chat-9B ranks strongly on selected generation-style tasks, but ranks lower on General_Text and regression tasks. NatureLM-8x7B does not show consistent gains across task groups. These results indicate that domain-specific fine-tuning may improve performance on selected task formats, while reducing robustness across broader general or out-of-domain settings.

#### 2.2.1 Classification tasks

**Figure 2:**
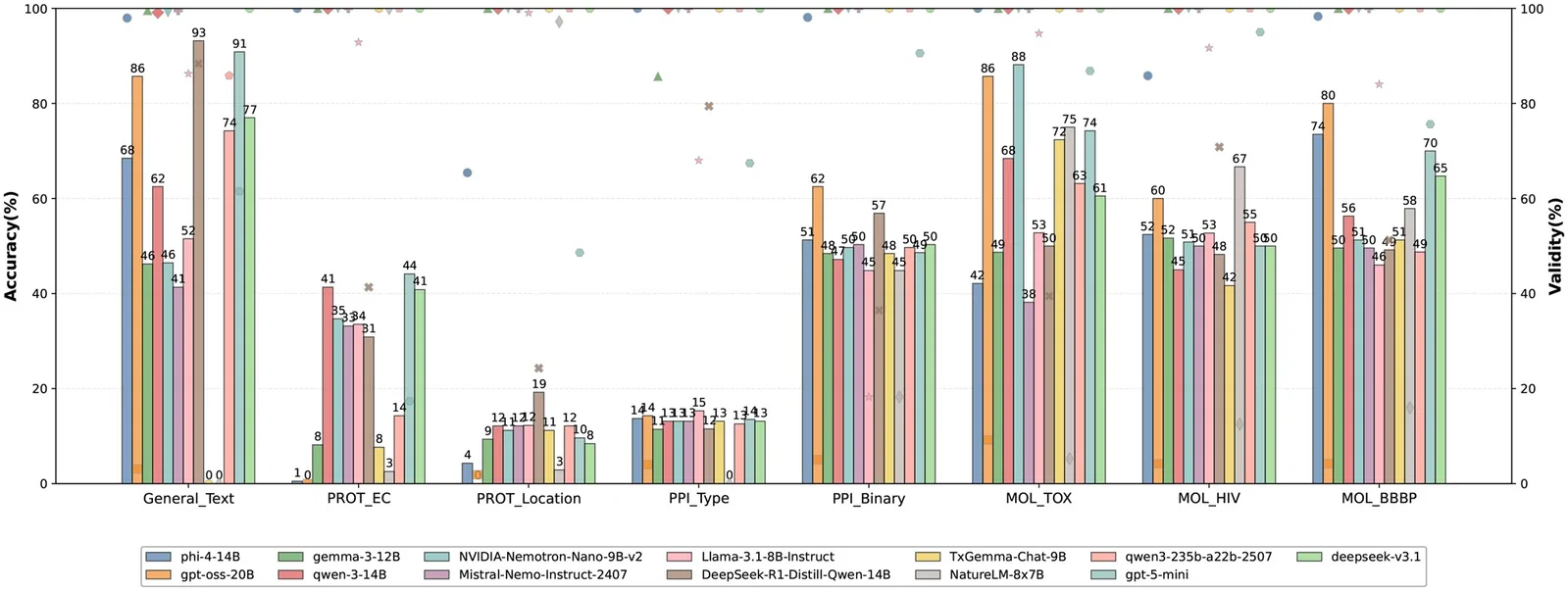
Performance of 13 LLMs among eight classification tasks. The prediction accuracy are displayed with bars based on the left axis. The markers represent model output validity based on the right axis. Larger-parameter models rank among the top performers in overall accuracy.

Firstly, we analyze model performance across eight classification tasks, evaluated in terms of prediction accuracy and output validity. As Fig. 2 shows, models achieve higher performance on text-only (General_Text) or easy classification tasks (MOL_BBBP and MOL_Tox), whereas performance declines for structurally complex or biologically nuanced tasks such as protein–protein interaction type prediction. Specifically, due to the deep fine-tuning process of NatureLM-8x7B, its accuracy on some tasks is zero as the fine-tuning process did not learn it. Large-parameter models, including Qwen3-235b-a22b-2507 and DeepSeek-v3.1, consistently rank among the top performers in overall accuracy. Medium-scale models such as Qwen-3-14B and Gemma-3-12B exhibit greater fluctuations and reduced robustness.

As for output validity (indicated with markers on Fig. 2), we measure whether model outputs conform to the required answer format and produce parsable predictions. Validity generally remains high for the majority of models, while certain models such as GPT-oss-20B demonstrate reduced validity in complicated tasks. Notably, some high-accuracy models such as Phi-4-14B occasionally show reduced validity in specific categories, suggesting that raw predictive capability does not necessarily guarantee strict adherence to output constraints. This distinction underscores the importance of jointly evaluating semantic correctness and formatting reliability in LLM benchmarking.

#### 2.2.2 Regression tasks

**Figure 3:**
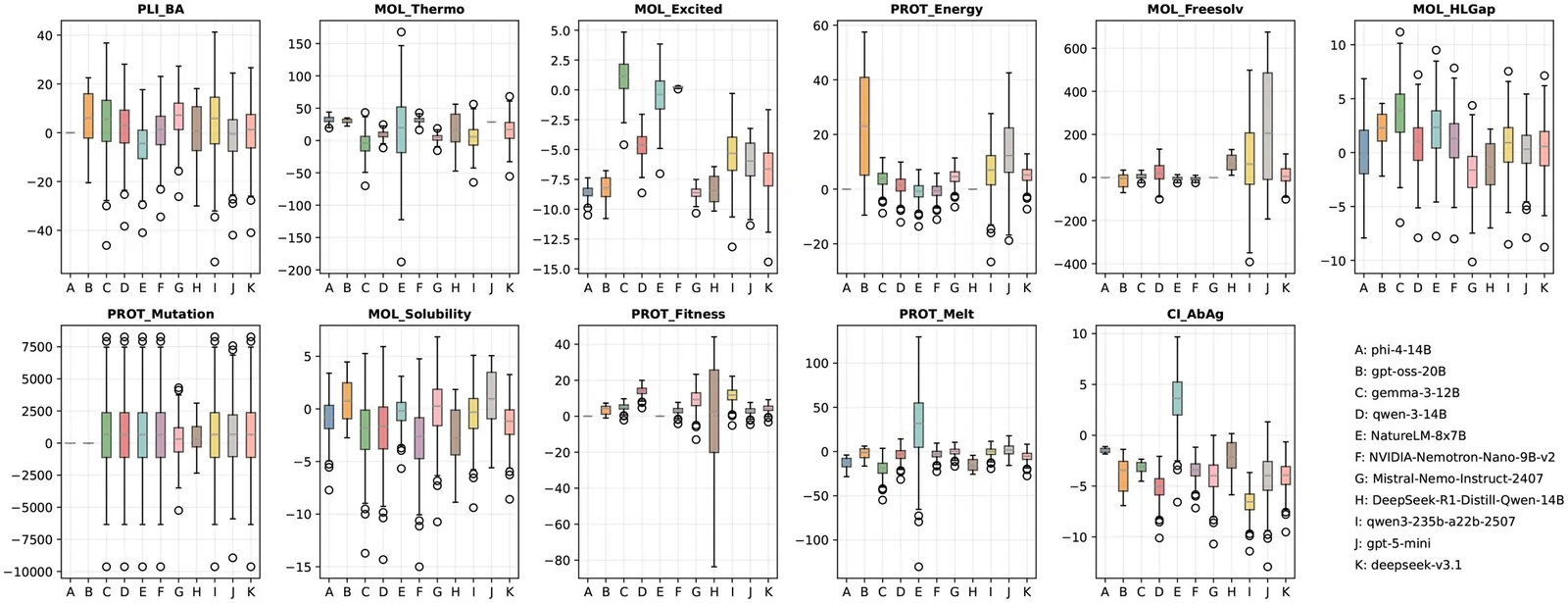
The distributions of prediction results on eleven regression tasks. By calculating the difference between each model’s predictions and the ground-truth labels across different samples for each task, the mean and variance are computed, from which the boxplot in each subplot is generated.

Figure 3 presents model performance across eleven bio-molecular regression tasks spanning molecular thermodynamics, quantum properties and protein mutational effects. Overall performance distribution across all tasks exhibits substantial variability. MOL_Thermo task requires quantitative prediction of thermodynamic properties from molecular SMILES, where small structural differences can lead to non-linear property variation. PROT_Mutation evaluates mutation-related protein properties from sequence-based inputs, requiring sensitivity to sequence context and long-range dependencies.

**Figure 4:**
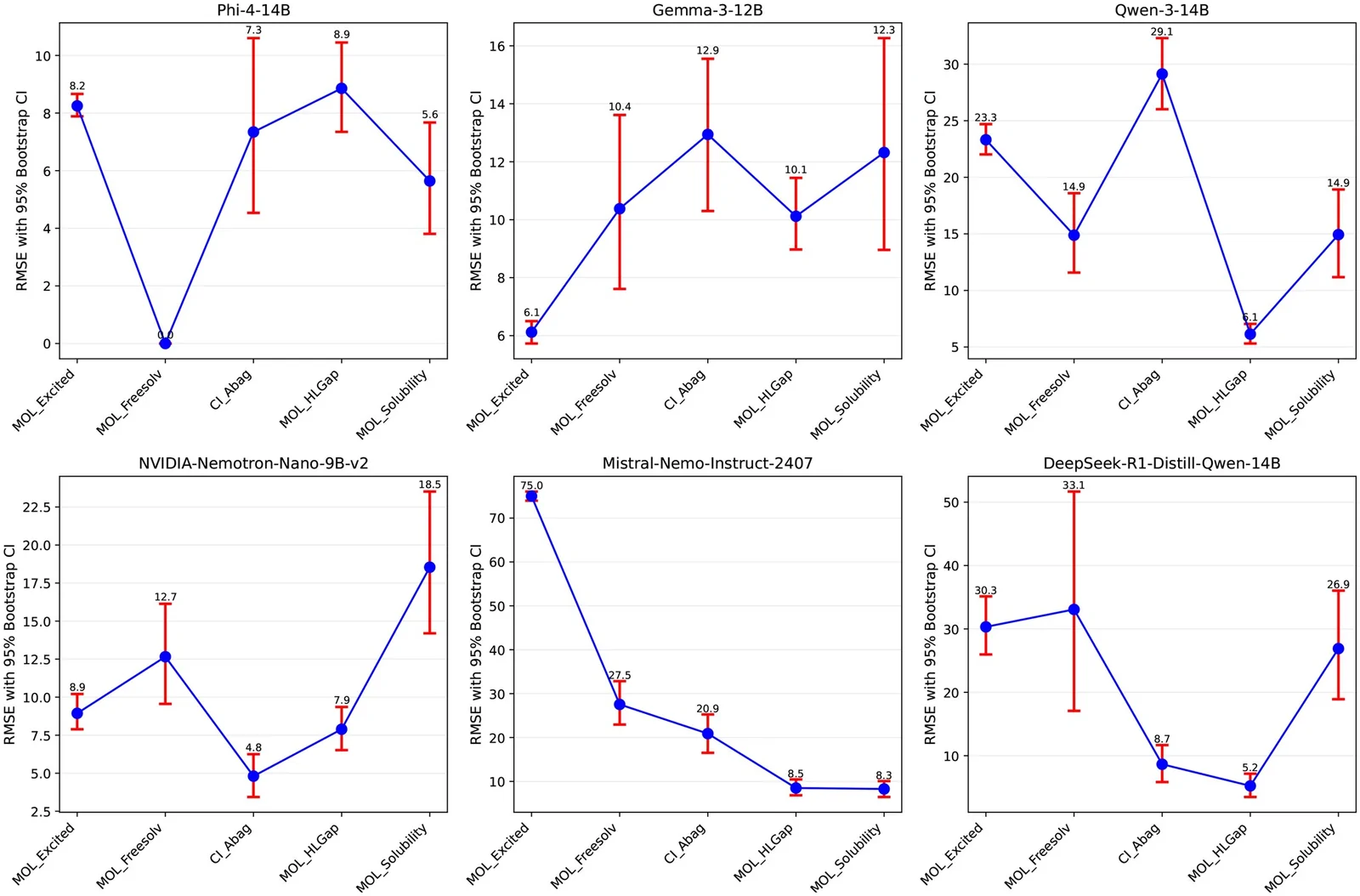
Bootstrakp-based confidence interval analysis. Several representative model-task pairs are analyzed. Tasks such as MOL_HLGap exhibit relatively narrow confidence intervals, indicating robust evaluation outcomes.

After parsing, Llama-3.1-8B-Instruct and TxGemma-Chat-9B models are unable to produce prediction results on any tasks, and are therefore not shown in Fig. 3. All models perform poorly on two extremely difficult tasks (MOL_Thermo and PROT_Mutation), indicating that future work should investigate ensemble methods to better handle the challenging long-sequence regression tasks prevalent in bio-molecular domains. To further strengthen the robustness of our evaluation, we additionally performed bootstrap-based confidence interval (CI) analysis on several representative model–task pairs as shown in Fig. 4. In general, tasks with larger RMSE values tend to exhibit wider confidence intervals. Among the models, NVIDIA-Nemotron-Nano-9B-v2 generally exhibits relatively narrow confidence intervals, indicating stable predictions under bootstrap resampling.

#### 2.2.3 Generation tasks

**Figure 5:**
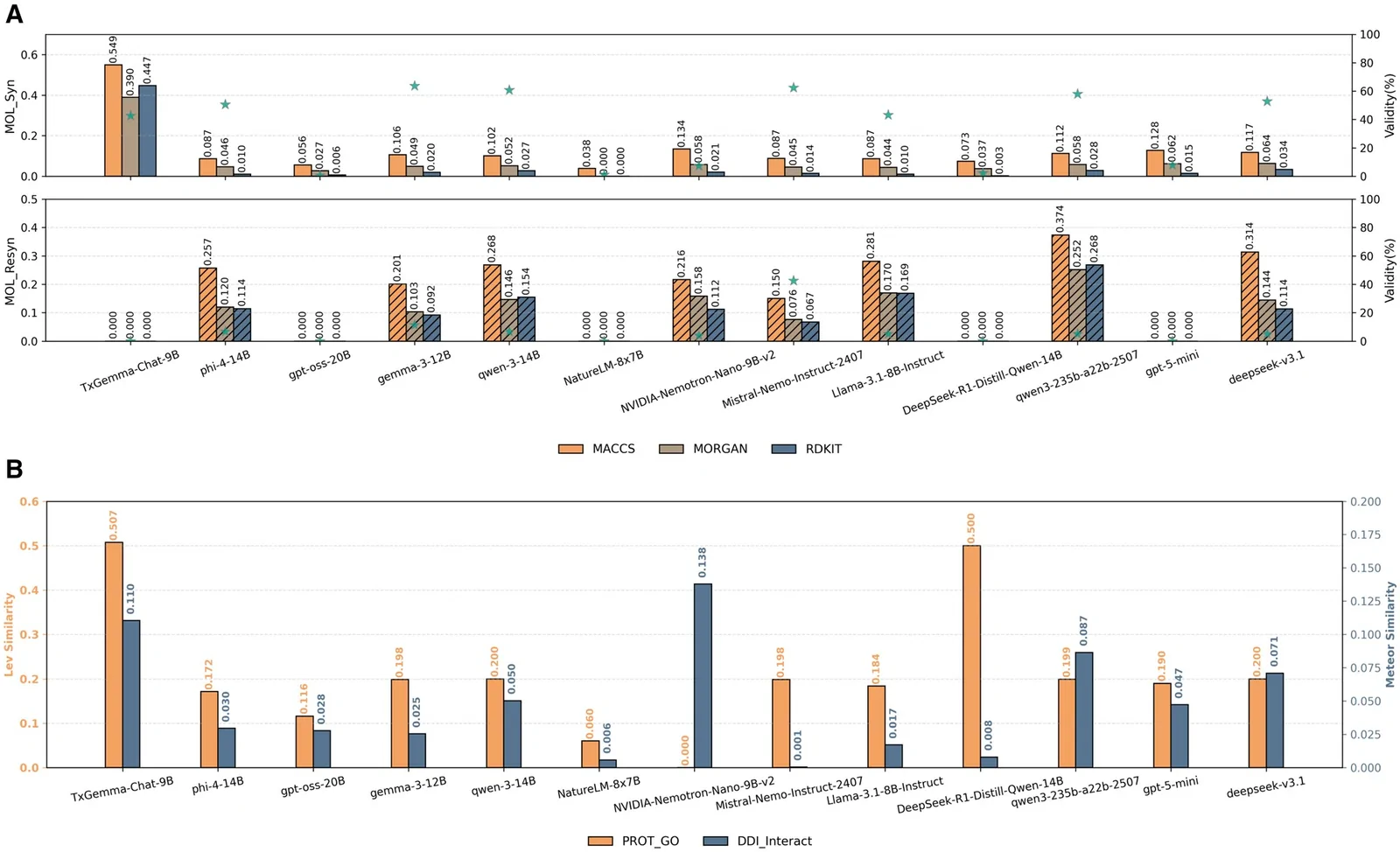
Model performance on generation tasks. Higher values indicate better model performance. (A) For MOL_Syn and MOL_Resyn tasks, fingerprint similarities of the prediction results were calculated using three methods: MACCS, MORGAN, and RDKIT. These values are displayed as bars corresponding to the left *y*-axis. The markers that represent the validity of the output results correspond to right *y*-axis. (B) The results for PROT_GO and DDI_Interact were evaluated with Levenshtein similarity and Meteor similarity metric, respectively.

There are seven generation tasks in our benchmark, Fig. 5 presents a comparative analysis of model behavior on four tasks. As for other three extremely hard tasks (PROT_Conserve, PROT_Location and PROT_Fold), all models are unable to complete, so the results are not shown. As for MOL_Syn task, TxGemma-Chat-9B demonstrates significant advantage over other models, based on the fingerprint similarity results using MACCS, MORGAN and RDKIT calculation methods. The results of other models show relatively minor differences. Furthermore, the fingerprint result distributions obtained from three calculation methods are consistent. Similarly, on PROT_GO and DDI_Interact tasks (panel b), TxGemma-Chat-9B demonstrates leading performance, highlighting the advantage of this model’s exceptional capabilities on generative tasks after fine-tuning with domain knowledge.

On the MOL_Resyn task, the overall performance of all models is mediocre. Among them, the Qwen3-series models achieves the best results. Five models including NatureLM-8x7B and DeepSeek-R1-Distill-Qwen-14B, are completely unable to generate valid molecules for the task. Notably, DeepSeek-R1-Distill-Qwen-14B, which was fine-tuned with chain-of-thought (CoT) based on Qwen-3-14B, shows weaker performance than its baseline model on this task. This result suggests that CoT-style fine-tuning does not necessarily improve performance on all bio-molecular generation tasks within our benchmark.

### 2.3 Tool capability comparison

**Figure 6:**
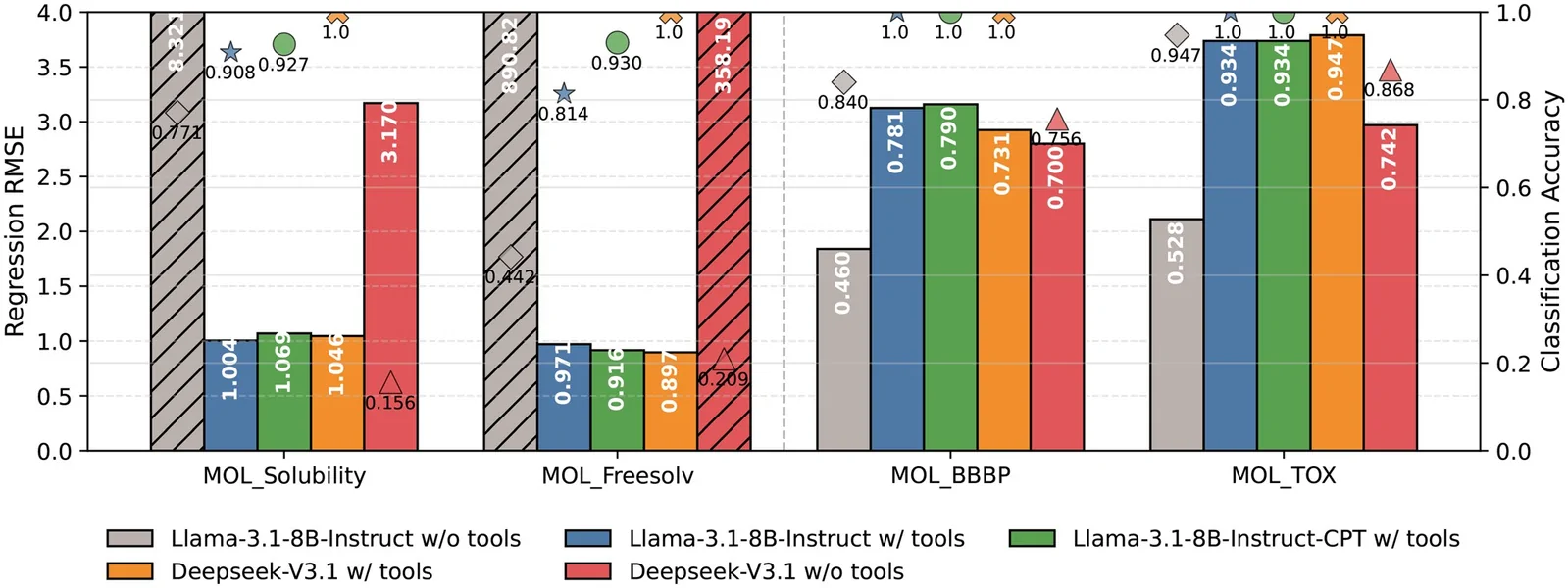
LLM performance with and without tool integration across four representative tasks. (left) For regression-based tasks (MOL_Solubility and MOL_Freesolv), performance is evaluated using RMSE, where lower value bars indicate better performance. The dashed bars indicate values exceeding the coordinate range, and the actual value is displayed with white numerical text. (right) For classification tasks, performance is measured using accuracy. The markers represent model output validity.

We compare benchmark performance between single LLMs using direct prompt and agentic workflows with tool integration. Figure 6 exhibits the performance of two different backbone models (DeepSeek-v3.1 and Llama-3.1-8B) on two regression and two classification tasks. Firstly, across all tasks and regardless of which model, tool-enabled configurations exhibit greater numerical stability and improved overall performance. The most striking effect is observed in regression tasks, where disabling tools leads to extreme error values, suggesting that unconstrained language generation is insufficient for precise quantitative estimation. Classification tasks show smaller but consistent performance drops without tools, implying that symbolic reasoning alone partially supports binary decision-making but lacks reliability.

Secondly, we compare the effect of continual pretraining (CPT). Llama-3.1-8B-Instruct-CPT is trained with sufficient bio-molecular knowledge. CPT introduces slight improvement in both regression and classification tasks. This indicates that domain-specific CPT enhances the model’s ability to interact effectively with structured chemical inputs and tool outputs. Finally, comparing the results of models of different parameter scales, it is counter-intuitive that although DeepSeek-V3.1 has over 50 times more parameters than LLama-3.1-8B, their overall performance is similar, outperforming each other in two tasks respectively.

These observations provide practical guidance on when LLMs and specialized models are more appropriate. Direct LLM prompting is more suitable for tasks involving natural-language interpretation, especially when the output space is categorical or text-based. In contrast, specialized computational tools are more reliable for calibrated numerical prediction and ADMET-like property estimation.

### 2.4 Ablation study of experiment settings

**Figure 7:**
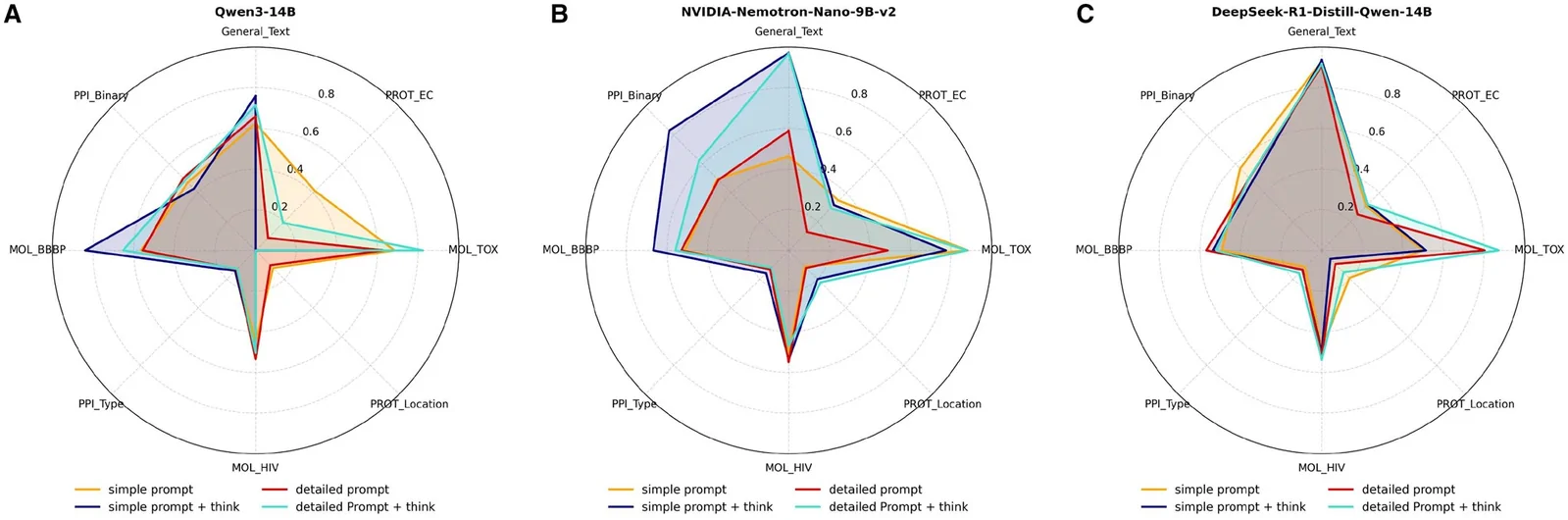
Model behavior under different input prompt complexity and think mode. (a) Qwen3-14B, (b) NVIDIA-Nemotron-Nano-9B-v2, and (c) DeepSeek-R1-Distill-Qwen-14B performance across different prompt settings and think mode configurations, with performance is evaluated with accuracy. Detailed prompt with more domain knowledge guidance generally exhibits better performance, and model performance under think mode varies depending on the task. NVIDIA-Nemotron-Nano-9B-v2 shows higher accuracy and sensitivity to different experiment settings.

We also examine model sensitivity to different prompts and think mode as minor variations in phrasing, formatting, or instruction style may lead to performance fluctuations. Qwen3-14B, NVIDIA-Nemotron-Nano-9B-v2 and DeepSeek-R1-Distill-Qwen-14B are selected because they support think mode. Simple and detailed prompt template are described in Section 4. Overall, NVIDIA-Nemotron-Nano-9B-v2 (Fig. 7) shows stronger sensitivity and variability to different think mode and input prompt than Qwen3-14B. And model performance on hard tasks such as PPI_Binary can be improved by think process. On most tasks, DeepSeek-R1-Distill-Qwen-14B’s sensitivity to prompt is low, with only significant differences observed on MOL_TOX task. These results indicate that when using LLM to complete domain-specific tasks, more domain knowledge guidance should be given, and whether to use think mode needs to be flexibly adjusted according to the difficulty of tasks.

### 2.5 LLM Reasoning ability in scientific problem

To investigate how LLMs solve scientific problem, we analyze their CoT reasoning processes. Beyond final-answer accuracy, this analysis focuses on whether intermediate reasoning reflects a principled understanding of biomolecular mechanisms. A representative example arises from molecular solvation free energy prediction task as follows. At first glance, this reasoning appears chemically sound. However, the reasoning processing contains some contradictions and chemical inconsistencies: If the molecule is fused and contains two ketones, it is more likely a naphthoquinone derivative or a dione, but not similar to acetanilide. Similar behavior is observed across task types and models. In generative tasks such as forward synthesis, models produce fluent and domain-relevant rationales, but lack a verifiable linkage to the governing chemical processes. These observations indicate that LLMs solve scientific problems primarily through a combination of pattern recognition, abstraction, and heuristic reasoning, rather than through explicit modeling of underlying mechanisms.

This issue is not limited to small-molecule reasoning. In subcellular location prediction task, reasoning process of Qwen3-14B is given below. Although model uses plausible terms, there is a logical conflict in the deduction. By acknowledging that the sequence has transmembrane domains but completely ruling out golgi targeting, the original analysis overlooks how membrane proteins are synthesized. If the N-terminus acts as a signal-anchor, the protein must be co-translationally translocated into the endoplasmic reticulum membrane first.

**Output example of DeepSeek-R1-Distill-Qwen-14B under think mode:** 

Okay, so I need to predict the free energy of a molecule given its SMILES notation. The SMILES provided is c1ccc2c(c1)C(=O)c3ccccc3C2 = O. Hmm, let me try to figure this out **step by step**.

** First**, [… Omitted for brevity …]

** Putting this together**, the molecule might have **two benzene rings connected in a fused manner**, with two ketone groups and an amine substituent. This structure could be **similar to acetanilide** or something more complex, perhaps a beta-lactam or another bicyclic compound.

**Output example of Qwen-14B under think mode:** 

The given protein sequence contains hydrophobic regions and potential transmembrane domains, which are often characteristic of proteins localized to the plasma membrane (class 3).

Additionally, the sequence does not show strong signals for nuclear localization (like a nuclear localization signal), mitochondrial targeting (like a presequence), or endoplasmic reticulum Golgi targeting (like a signal peptide).

Based on these features, the most likely subcellular localization is the plasma membrane.

## 3 Discussion

The cross-scale bio-molecular benchmark introduced in this work provides a comprehensive evaluation framework for assessing large language models on biologically-relevant tasks spanning multiple scales of molecular complexity. Through systematic evaluation of 13 state-of-the-art models, we have elucidated several important insights regarding model capabilities, architectural influence, and the value of tool integration in the bio-molecular domain.

Our ranking analysis reveals that NVIDIA-Nemotron-Nano-9B-v2, which employs a hybrid mamba-attention architecture, achieves the best overall performance across BioMol-LLM-Bench. This suggests that hybrid architecture appears particularly well-suited for processing the long-range dependencies inherent in bio-molecular sequences. Poor performance across all models on 2 challenging tasks (MOL_Thermo and PROT_Mutation) highlights the difficulty of long-sequence regression in bio-molecular applications and motivates exploration of model optimization. Models that underwent extensive domain-specific fine-tuning, such as TxGemma-Chat-9B, show strong performance on a subset of tasks but lower performance on general text understanding in our evaluation. This pattern is consistent with a specialization–generalization trade-off, but should be interpreted as an observation from the evaluated models rather than a universal consequence of SFT. Through the analysis of reasoning process, we found that LLMs may arrive at the correct answer but with wrong mechanistic reasons.

Overall, the consistently high performance on lower-level tasks (L0–L1 levels) suggests that current LLMs have developed robust representations for small molecular language when expressed in natural language format. However, the marked performance degradation on L3-level tasks involving protein-protein interactions indicates a limitation of the evaluated models in reasoning about relationships between molecules under the current benchmark setting. The observation that tool-enabled configurations outperform direct prompting in the selected tool-supported tasks supports a hybrid setting where LLMs help organize task instructions and tool arguments, whereas specialized tools handle quantitative computations. Because BioMol-LLM-Bench is constructed from public bio-molecular resources, possible pretraining overlap should be considered when interpreting model performance. For this reason, we focus on comparative trends across task types, model architectures, prompting settings, and tool-enabled configurations within the same benchmark, rather than treating single-task scores as direct evidence of out-of-distribution generalization. In conclusion, the cross-scale bio-molecular benchmark provides both a standardized evaluation methodology and empirical insights that are promising to guide future development of language model design for bio-molecular discovery.

## 4 Methods

### 4.1 Benchmark construction

We propose a comprehensive benchmark dataset specifically tailored for evaluating the capabilities of LLMs in molecular sciences. The source dataset is carefully derived from multiple high-quality sources to ensure diversity and reliability. Moreover, tool calling API and unified evaluation pipeline are integrated to facilitate efficient model comparison.

1. *Diverse Raw Open-Source Data Sources*. The foundation of the benchmark draws from a variety of raw open-source data, combining domain-specific benchmarks (e.g. MoleculeNet Wu *et al.* (2018) suites including QM9 for quantum properties and Tox21 for toxicity) with general semantic understanding benchmarks (e.g. biomolecule-related questions in MMLU-Pro and AI2ARC). Detailed descriptions of tasks are given in Table 1 and Supplementary Table S1. This integration ensures the dataset to capture both specialized molecular tasks and broader chemical knowledge.
2. *Standardized Data Processing Workflow.* To maintain consistency and quality, a rigorous standardized workflow is applied.
  - *Filtering*. Invalid, incomplete, or noisy entries were removed before benchmark construction. For molecular tasks, RDKit was used to check molecular validity and remove invalid SMILES strings or duplicated molecular records. Moreover, DeepSeek-v3.1 was used as an auxiliary verifier following the prompt below, judging whether each instance was related to small-molecule or protein science and assigning a confidence score.
*Prompt for sample filtering:* You are an expert in life science.
    Please carefully analyze the following question and judge that whether (Yes or No) the question related to small molecule and protein science, and give the confidence score (between 0 and 1) about your answer.
     Question: {input_text}
     Please answer in exactly the following format:
     Answer:
     Confidence:
  - *Cleaning.* Molecular representations were normalized into canonical SMILES format, and inconsistent or ambiguous labels were resolved according to the original dataset annotations. For protein-related tasks, protein sequences are standardized to uppercase.
  - *Merging.* Data from multiple sources were integrated after task-specific normalization. To avoid overlap across merged datasets, duplicate or highly similar entries were removed based on canonical molecular representations and sequence identifiers.
  - *Length casting.* Natural language text with excessive byte counts (more than 1000) or protein sequences longer than 500 characters were filtered out to reduce computational complexity and avoid excessively long prompts.
  - *Sampling.* Stratified sampling was applied to balance molecular complexity and property distributions. For classification tasks, samples were selected according to label categories; for regression tasks, continuous labels were sampled to preserve coverage of the property range; and for paired-input tasks, sampling was performed at the pair level.
  - *Metadata supplement.* For complicated tasks such as protein ligand interaction prediction, additional protein and molecule structure information are provided.
3. *Coverage of Diverse Tasks Across Molecular Scales.* The benchmark encompasses 4 levels of tasks according to input complexity.
  - *L0: General bio-molecular knowledge understanding.* Input data type of questions in this level only contains natural language.
  - *L1: Small-molecule level tasks.* Focusing on small molecule property prediction including chemical and physiological property. Both natural language and SMILES representation of molecules are provided.
  - *L2: Large-molecule level task.* Focusing on tasks related to protein function and structure. Input data modalities include natural language and protein sequence.
  - *L3: Multiple-molecules level task.* Incorporating challenging real-world tasks, such as antibody-antigen binding prediction, with more input data types including molecule SMILES, protein sequence, and natural language.
    Processed datasets for each task were stored in standard JSON format, with columns including canonical SMILES, protein sequence, task-specific labels, and metadata.
4. *Automated Result Parsing and Evaluation Pipeline.* To enable fair and reproducible comparison of LLMs, we proposed automatic output parsing protocol, through which the extracted type-specific answers of different models were obtained, and the performance of the model is further evaluated through standard task-specific evaluation metrics.
  - Classification tasks contain MOL_TOX, MOL_HIV, MOL_BBBP, PPI_Binary, PROT_EC, PROT_Location, General_Text and PPI_Type. Model performance is evaluated through accuracy and answer-type validity.
  - Regression tasks contain MOL_HLGap, MOL_Thermo, MOL_Excited, CI_AbAg, PLI_BA, PROT_Mutation, PROT_Fitness, PROT_Energy, PROT_Melt, MOL_Freesolv and MOL_Solubility. Model performance is evaluated through RMSE, MEAN, STD and answer-type validity.
  - Generation tasks contain MOL_Resyn, MOL_Syn, PROT_GO, PROT_Conserve, PROT_SSC, PROT_Fold and DDI_Interact. Model performance is evaluated through similarity metrics such as MACCS, RDK, MORGAN, Meteor and Levenshtein distance.
5. *Tool calling API.* Candidate tools were sourced exclusively from ToolUniverse Gao *et al.* (2025a), which standardizes tool specifications in JSON format (including name, description, parameters, and execution endpoints). The selected tools for this evaluation were:
  -
ADMETAI_predict_solubility_lipophilicity_hydration : A graph neural network-based platform for rapid prediction of ADMET properties across large chemical libraries. The output values of Solubility_AqSolDB and HydrationFreeEnergy_FreeSolv property represent results of MOL_Solubility and MOL_Freesolv task, respectively.
  -
ADMETAI_predict_BBB_penetrance : used to execute MOL_BBBP task, representing with BBB_Martins property value.
  -
ADMETAI_predict_stress_response : used to execute MOL_TOX task, representing with SR−ARE toxicity value.

In the tool-enabled setting, tool use was evaluated in a controlled task-specific manner. The tool-supported tasks and candidate tools were predefined according to the target property of each task. For each tool-supported task, the model was provided with the task prompt, the molecular input, the available tool name, a short tool description, and the required argument format. The model was then asked to generate a structured tool call using the corresponding arguments. Invalid tool names, missing arguments, unparsable calls, or invalid SMILES inputs were counted as failed tool-use cases.

### 4.2 LLM evalution

1. *Model Selection and Deployment.* Both open-source and closed-source LLMs were selected to enable transparent and reproducible deployment. The primary models evaluated were listed in Table 3. The parameter scale, model architecture, and thinking mode of these models vary. For open-source models, we deployed them with *vllm*  Kwon *et al*. (2023). For closed-source models, batch testing was conducted by calling the API interface (*openai*.*client*) with equivalent prompts. Temperature was set to 0.7, and top-p was fixed at 1.0 by default. Various comparative experiments and analyses were conducted, including:
  - The performance of general-purpose LLMs and domain-specific fine-tuned models on BioMol-LLM-Bench.
  - Based on the same backbone model, compare model performance before and after CoT fine-tuning (Qwen-3-14B vs. DeepSeek-R1-Distill-Qwen-14B).
  - Compare the performance of models with different parameter scales.
  - The performance of different architecture models (dense transformer, MoE, Mamba) under similar parameter scales.
  - The impact of input prompt complexity, think mode, and other configurations.
  - The impact of external tool assistance on model performance.
2. *Prompt definition.* Consistent prompts were designed to standardize input for general-purpose LLMs. Prompts were stored as reusable templates in a configuration file. For each task, a natural language prompt template was defined, incorporating the molecular representation and task-specific instructions. Models were prompted in a standardized zero-shot manner. To compare the prompt complexity influence on model performance, we also define the simplified prompt template for each task.

**Table 3:** Overview of evaluated models.

Model	#Params	Open	Domain-FT	Architecture
DeepSeek-v3.1 DeepSeek-AI (2025)	685B	✓	✗	MoE
Qwen3-235b-a22b-2507 Bai *et al.* (2023)	235B	✓	✗	MoE
GPT-5-mini OpenAI (2025)	/	✗	✗	MoE
NatureLM-8x7B Xia *et al.* (2025)	56B	✓	✓	MoE
TxGemma-Chat-9B Wang *et al.* (2025)	9B	✓	✓	D.T.
Phi-4-14B Abdin *et al.* (2024)	14B	✓	✗	D.T.
GPT-oss-20B Agarwal *et al.* (2025)	20B	✓	✗	D.T.
Gemma-3-12B Gemma Team *et al.* (2024)	12B	✓	✗	D.T.
Qwen-3-14B Bai *et al.* (2023)	14B	✓	✗	D.T.
NVIDIA-Nemotron-Nano-9B-v2 Basant *et al.* (2025)	9B	✓	✗	Mamba
Mistral-Nemo-Instruct-2407 The Mistral AI Team (2024)	12B	✓	✗	D.T.
Llama-3.1-8B-Instruct Touvron *et al.* (2023)	8B	✓	✗	D.T.
DeepSeek-R1-Distill-Qwen-14B Guo *et al.* (2025)	14B	✓	✗	D.T.

The table lists 13 language models evaluated, models vary in the number of parameters (#Params), open-source availability (Open), whether the model has undergone domain-specific fine-tuning (Domain-FT), and the underlying architectural type (Architecture). MoE denotes Mixture-of-Experts architecture, while D.T. refers to Dense Transformer. Parameter counts marked with “/” indicate undisclosed model sizes.

**Simple prompt template for MOL_Freesolv task:** 

Context: Given the [SMILES] sequence of a molecule, your task is to predict its free energy.

 [SMILES]: {}

 Your response should be in the following format:

 Answer: {your answer, representing with a floating-point number.}

**Detailed prompt template for MOL_Freesolv task:** 

Instruction: The solvation free energy of a molecule is a thermodynamic measure of how favorably a molecule dissolves in a solvent (typically water).

 Context: Given the [SMILES] sequence of a molecule, your task is to predict its free energy.

  [SMILES]: {}

 Your response should be in the following format:

 Explanation: {your explanation for your answer, this is optional.}

 Answer: {your answer, representing with a floating-point number.}

 Confidence: {your confidence score between 0% and 100% for your answer.}

For fine-tuned domain-specific models such as NatureLM-8x7B and TxGemma-Chat-9B, since they have predefined fine-tuning templates, we construct prompts for each task by referencing the original template.

**Prompt template for NatureLM-8x7B:** 

Instruction: Predict solvation free energy of the molecule. {}

Response:

**Prompt template for TxGemma-Chat-9B:** 

Instructions: Answer the following question about molecular property.

Context: The solvation free energy of a molecule is a thermodynamic measure of how favorably a molecule dissolves in a solvent (typically water).

Question: Given the [SMILES] sequence of a molecule, predict its free energy.

[SMILES]: {}

Answer:

## Supplementary Material

btag550_Supplementary_Data

## Data Availability

Source code is available at https://github.com/AI-HPC-Research-Team/BioMol-LLM-Bench, and the benchmark dataset is available at https://github.com/AI-HPC-Research-Team/BioMol-LLM-Bench/tree/main/dataset. The code is released under Apache-2.0 License.
