# Supplementary material for: The limits of bio-molecular modeling with large language models: a cross-scale evaluation
Source: Bioinformatics. 2026 Jul 23;42(8):btag550. doi: 10.1093/bioinformatics/btag550 (PMC13457088; doi:10.1093/bioinformatics/btag550)
Supplement: Supplementary file 1 [file btag550_supplementary_data.pdf]

Supplementary Tables

Supplementary Table S1: Detailed descriptions of benchmark task.

Level	Task (abbr.)	Description
L0	General_Text	Domain knowledge understanding with text-input only.
L1	MOL_Solubility	The solubility of a molecule refers to its ability to dissolve in a solvent (typically water) to form a homogeneous solution.
	MOL_Freesolv	The solvation free energy of a molecule is a thermodynamic measure of how favorably a molecule dissolves in a solvent (typically water).
	MOL_Syn	Predict the forward synthesis production of two molecules.
	MOL_Resyn	Given production, predict the reactant molecules.
	MOL_Excited	E1 property of a moleculer refers to the energy of lowest singlet excited state relative to the ground state. Predict the energy of lowest singlet excited state relative to ground state of molecule in eV units.
	MOL_Thermo	Predict the heat capacity of molecule in kcal/mol/K scale.
	MOL_BBBP	The BBBP (Blood-Brain Barrier Penetration) property represents whether a molecule can cross the blood-brain barrier.
	MOL_HIV	The HIV replication prevention property refers to a molecule's ability to inhibit one or more steps in the HIV replication cycle, thereby preventing viral proliferation.
	MOL_HLGap	The HOMO-LUMO gap (Highest Occupied Molecular Orbital - Lowest Unoccupied Molecular Orbital gap) represents energy difference between HOMO and LUMO (measured in eV or kcal/mol).
	MOL_TOX	The SR-ARE (Sulfonamide Reactive - Aryl Ester) toxicity refers to the adverse effects caused by molecules containing sulfonamide and aryl ester functional groups.
L2	PROT_Melt	The melting point of a protein is the temperature at which 50% of the protein unfolds, marking the midpoint of the transition from the folded (native) state to the unfolded (denatured) state.
	PROT_Energy	The stability of a mutated protein sequence represents the free energy difference between mutated sequence and its native sequence.
	PROT_Fitness	The fitness landscape represents how mutations affect functionality (e.g., enzymatic activity, binding affinity, stability) of a protein.
	PROT_Mutation	The mutation score of mutated protein quantifies the functional/structural impact of a small fraction of amino acid changes.
	PROT_Conserve	The conservation score measures how evolutionarily conserved a residue across homologs (reflects purifying selection).
	PROT_Location	The subcellular localization class of a protein refers to its specific compartment or organelle within a cell where it predominantly resides and functions.
	PROT_EC	The Enzyme Commission (EC) system classifies enzymes into a hierarchical numbering system (e.g., EC 1.1.1.1 for alcohol dehydrogenase) based on their catalytic reactions. The task is to predict first-level EC class number of protein.
	PROT_GO	The Gene Ontology (GO) describes protein functions in three categories: Molecular Function (MF), Biological Process (BP) and Cellular Component (CC). The task is to predict the Molecular Function (MF) properties of protein.
	PROT_SSC	Secondary structure classification typically follows the DSSP (Define Secondary Structure of Proteins) standard, which categorizes each amino acid of the protein into distinct classes.
	PROT_Fold	Predict the 3D structure of protein.
L3	PPI_Type	Predict the PPI interaction types between two proteins.
	PPI_Binary	The task of predicting whether two proteins interact or not.
	DDI_Interact	Predict the DDI interaction type between two drugs.
	PLI_BA	Predict the PLI binding affinity between protein and ligand.
	CI_AbAg	Predict the AbAg binding affinity between antibody (heavy and light chain) and antigen.

Supplementary Table S2: **Task-group average ranking of domain-specific fine-tuned models.** Lower values indicate better performance. Domain-FT models include TxGemma-Chat-9B and NatureLM-8x7B. Non-Domain-FT denotes the best task-group average rank among models without domain-specific fine-tuning.

Task group	Included tasks	TxGemma-Chat-9B	NatureLM-8x7B	Non-Domain-FT	Interpretation
General text	General_Text	12.00	13.00	1.00	Domain-FT models rank lower on general text understanding.
Classification	General_Text, PROT_EC, PROT_Location, PPI_Type, PPI_Binary, MOL_TOX, MOL_HIV, MOL_BBBP	7.25	12.25	3.88	Domain-specific fine-tuning does not uniformly improve classification tasks.
Regression	PLI_BA, MOL_Thermo, MOL_Excited, PROT_Energy, MOL_Freesolv, MOL_HLGap, PROT_Mutation, MOL_Solubility, PROT_Fitness, PROT_Melt, CI_AbAg	12.00	9.00	2.55	Domain-FT models remain weak on calibrated numerical prediction.
Generation	PROT_GO, DDI_Interact, MOL_Syn, MOL_Resyn	3.25	11.75	3.50	TxGemma-Chat-9B shows advantage on selected generation-style tasks.
Protein-level	PROT_EC, PROT_Location, PROT_Energy, PROT_Mutation, PROT_Fitness, PROT_Melt, PROT_GO	9.29	10.86	3.86	Domain-FT does not consistently improve protein-level tasks.
Interaction-level	PPI_Type, PPI_Binary, PLI_BA, DDI_Interact, CI_AbAg	7.60	10.60	2.40	Interaction-level tasks remain difficult for domain-specific model.
